# Supplementary figures and images for: Investigation on Purification of Saturated LiNO3 Solution Using Titanium Phosphate Ion Exchanger: Kinetics Study
Source: Int J Mol Sci. 2022 Nov 2;23(21):13416. doi: 10.3390/ijms232113416 (PMC9657845; doi:10.3390/ijms232113416)

Supporting Information

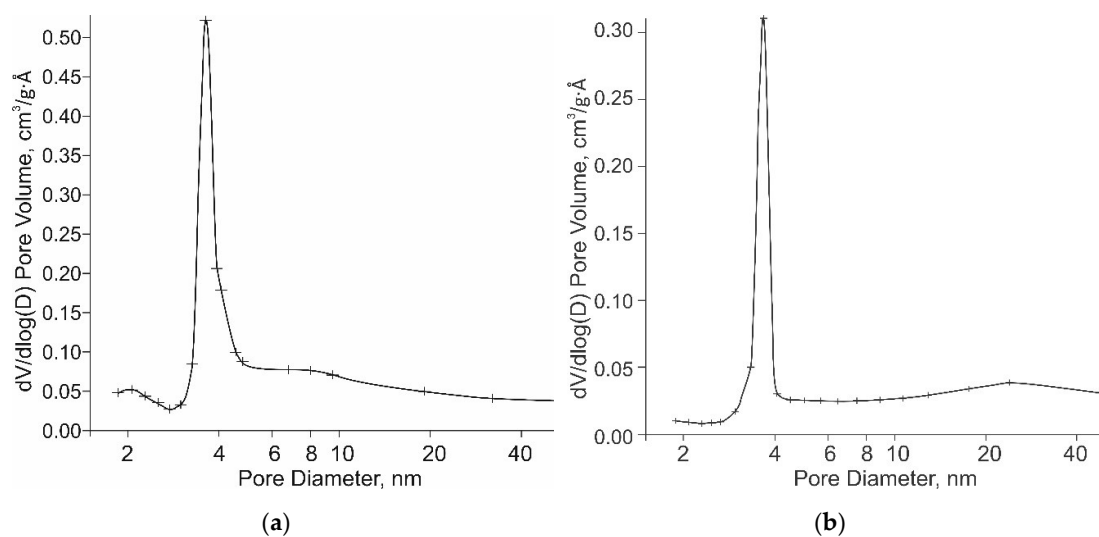

**Figure S1.** The pore size distribution plots for TiOP (a), and Li-TiOP (b).

Supplement: Supplementary file 1 [file ijms-23-13416-s001.zip › ijms-1971841-supplementary.pdf]
